# Coherence properties of focused X-ray beams at high-brilliance synchrotron sources

**DOI:** 10.1107/S1600577513023850

**Published:** 2013-11-02

**Authors:** Andrej Singer, Ivan A. Vartanyants

**Affiliations:** a Deutsches Elektronen-Synchrotron DESY, Notkestrasse 85, D-22607 Hamburg, Germany; b National Research Nuclear University ’MEPhI’, 115409 Moscow, Russia

**Keywords:** X-ray lenses, partial coherence

## Abstract

An analytical approach describing properties of focused partially coherent X-ray beams is presented. The method is based on the results of statistical optics and gives both the beam size and transverse coherence length at any distance behind an optical element. In particular, here Gaussian Schell-model beams and thin optical elements are considered. Limiting cases of incoherent and fully coherent illumination of the focusing element are discussed. The effect of the beam-defining aperture, typically used in combination with focusing elements at synchrotron sources to improve transverse coherence, is also analyzed in detail. As an example, the coherence properties in the focal region of compound refractive lenses at the PETRA III synchrotron source are analyzed.

## Abstract 2

An analytical approach describing properties of focused partially coherent X-ray beams is presented.

## 1. Introduction

While ultimate storage rings, being diffraction-limited X-ray sources, are still under development (Bei *et al.*, 2010 ▶), present third-generation synchrotrons are partially coherent sources (Vartanyants & Singer, 2010 ▶). The construction of these sources initiated developments of new research areas, which utilize partial coherence of the X-ray radiation. Most prominent among these techniques are coherent X-ray diffractive imaging (CXDI) (Vartanyants *et al.*, 2010 ▶; Chapman & Nugent, 2010 ▶; Mancuso *et al.*, 2010 ▶; Vartanyants & Yefanov, 2013 ▶) and X-ray photon correlation spectroscopy (XPCS) (Grübel & Zontone, 2004 ▶). In CXDI static real space images of the sample are obtained by phase retrieval techniques (Fienup, 1982 ▶), whereas in XPCS dynamics of a system are explored by correlation techniques (Goodman, 2007 ▶).

The key feature of all coherence-based methods is the interference of the field scattered by different parts of the sample. As such, spatial coherence across the sample is essential and understanding the coherence properties of the incoming X-ray beams generated at new generation synchrotron sources is of vital importance for the scientific community. A detailed knowledge of the coherence properties can even be used to improve the resolution obtained in the CXDI phase retrieval (Whitehead *et al.*, 2009 ▶).

**Figure 1:**
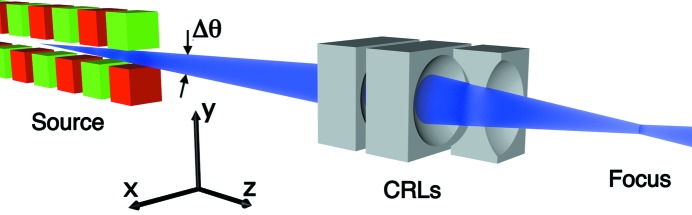
Partially coherent radiation is generated in the undulator and is focused by a stack of CRLs. Intensity and coherence properties of the focused radiation are considered. The last lens is cut to indicate the structure of a single CRL.

For scientific applications at the nanoscale, beam sizes from tens to hundreds of nanometers with high flux densities are required. These can be achieved by an effective use of focusing elements. Nowadays several techniques to focus X-ray beams at third- and fourth-generation sources are used, such as Kirkpatrick–Baez (KB) mirrors (Mimura *et al.*, 2010 ▶), Fresnel zone plates (Sakdinawat & Attwood, 2010 ▶), bent crystals in Bragg geometry (Zhu *et al.*, 2012 ▶) and compound refractive lenses (CRLs) (Snigirev *et al.*, 1996 ▶; Schroer *et al.*, 2003 ▶). A typical focusing scheme is shown in Fig. 1 ▶. Synchrotron radiation is generated in the undulator and a focusing element consisting of a stack of CRLs focuses the beam. In this paper we describe the propagation of partially coherent X-ray radiation through such a focusing system and determine its size and coherence properties at any position downstream from the CRL. Our results can be naturally generalized to other types of focusing elements such as Fresnel zone plates.

Synchrotron sources are generally considered as incoherent sources, since different electrons in the electron bunch radiate independently in the frame moving with the electrons. Due to relativistic effects, in the laboratory frame the radiation is confined to a narrow cone of angles 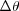 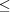 1/2γ (see Fig. 1 ▶), where γ is the Lorentz factor. This relativistic confinement implies an effective degree of transverse coherence at the source, as totally incoherent sources radiate into all directions (Goodman, 1985 ▶).

The transverse coherence area 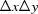 of a synchrotron source can be estimated from Heisenberg’s uncertainty principle (Mandel & Wolf, 1995 ▶), 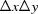 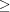 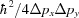, where 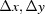 and 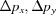 are the uncertainties in the position and momentum in the horizontal and vertical direction, respectively. Due to the de Broglie relation *p* = 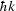, where *k* = 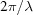, the uncertainty in the momentum 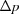 can be associated with the source divergence 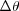, 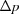 = 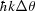, and the coherence area in the source plane is given by
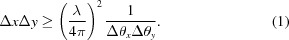
Substituting typical values of the source divergence at a third-generation synchrotron source (Balewski *et al.*, 2004 ▶) into equation (1), we find the minimum transverse coherence length at the source to be about a few micrometers. With the source sizes of tens to hundreds of micrometers (Balewski *et al.*, 2004 ▶), it is clear that present third-generation X-ray sources have to be described as partially coherent sources.

A useful model to describe the radiation properties of partially coherent sources is the Gaussian Schell-model (GSM) (Mandel & Wolf, 1995 ▶). This model has been applied for the analysis of the radiation field generated by optical lasers (Gori, 1980 ▶), third-generation synchrotron sources (see, for example, Howels & Kincaid, 1994 ▶; Vartanyants & Singer, 2010 ▶, and references therein) and X-ray free-electron lasers (Singer *et al.*, 2008 ▶, 2012 ▶; Roling *et al.*, 2011 ▶; Vartanyants *et al.*, 2011 ▶). The problem of propagation of partially coherent radiation through thin optical elements (OEs) in the frame of GSM has been widely discussed in optics (Turunen & Friberg, 1986 ▶; Yura & Hanson, 1987 ▶). However, the propagation of partially coherent radiation through the focusing elements with finite apertures has not been considered before. For X-ray beamlines at third-generation synchrotron sources such focusing elements are especially important. In this work we propose a general approach to describe propagation of partially coherent radiation through these beamlines.

The paper is organized as follows. We start with a short introduction to the optical coherence theory with special focus on third-generation synchrotron radiation sources in §2. §3 describes the propagation of partially coherent X-ray radiation through thin focusing elements. Diffraction-limited focus and infinite apertures are described in §4 and §5. In §6 coherence properties of the focused X-ray beams at PETRA III are analyzed. The paper is concluded with a summary and outlook.

## 2. Coherence: basic equations

### 2.1. Correlation functions and propagation in free-space

The theory of partially coherent fields is based on the treatment of correlation functions of the complex wavefield (Mandel & Wolf, 1995 ▶). The concept of optical coherence is often associated with interference phenomena, where the mutual coherence function (MCF)^1^

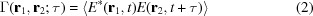
plays the main role. It describes the correlations between two complex values of the electric field 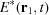 and 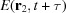 at different points 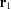 and 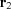 and different times *t* and 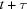. The brackets 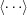 denote the time average.

When we consider propagation of the correlation function of the field in free space, it is convenient to introduce the cross-spectral density function (CSD), 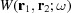, which is defined as the Fourier transform of the MCF (Mandel & Wolf, 1995 ▶),
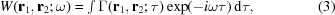
where ω is the frequency of the radiation. By definition, when the two points 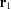 and 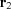 coincide, the CSD represents the spectral density of the radiation field,
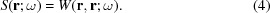
The normalized CSD is known as the spectral degree of coherence (SDC),
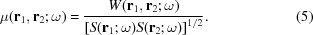
For all values of 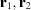 and ω the SDC satisfies 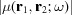 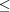 1. The modulus of the SDC can be measured in interference experiments as the contrast of the interference fringes (Singer *et al.*, 2008 ▶, 2012 ▶; Vartanyants *et al.*, 2011 ▶).

To characterize the transverse coherence properties of a wavefield by a single number, the global degree of transverse coherence can be introduced as (Saldin *et al.*, 2008 ▶; Vartanyants & Singer, 2010 ▶)
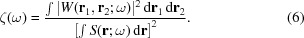
According to its definition the values of the parameter 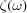 lie in the range 0 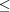 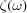 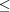 1, where 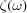 = 1 and 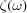 = 0 characterize fully coherent and incoherent radiation, respectively.

In the following we will apply the concept of correlation functions to planar secondary sources (Mandel & Wolf, 1995 ▶), where the CSD of the radiation field is given in the source plane at 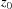 = 0 with the transverse coordinates **s**, 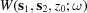. The propagation of the CSD from the source plane at 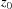 to the plane at a distance *z* from the source is governed by the following expression (Mandel & Wolf, 1995 ▶),
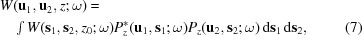
where 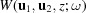 is the propagated CSD in the plane *z*, and 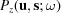 is the propagator. The integration is performed in the source plane. For partially coherent X-ray radiation at third-generation synchrotron sources it is typically sufficient to use the Fresnel propagator (Goodman, 2005 ▶), which is given by
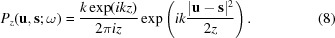



### 2.2. Gaussian Schell-model sources

The CSD of a GSM source positioned in the plane at 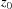 is expressed as (Mandel & Wolf, 1995 ▶)^2^

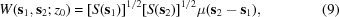
where the spectral density and the SDC in the source plane are Gaussian functions,
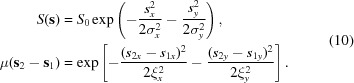
Here 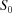 is a normalization constant, and the parameters 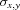 and 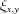 define the source size and transverse coherence length in the source plane in the *x*- and *y*-directions, respectively. Below all values are presented as root-mean-square (r.m.s.) values, if not stated differently.

The expression of the CSD function in the form of equation (9) is based on the definition of the SDC (5). In the GSM the main approximations are that the source is spatially uniform, *i.e.* 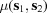 = 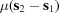, and all functional dependencies are described by Gaussian functions.

The CSD 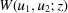 at the distance *z* from the source can be calculated using integration of (7) with the Fresnel propagator (8) (Mandel & Wolf, 1995 ▶)^3^

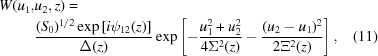
where 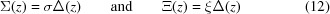
are the beam size and transverse coherence length at the distance *z* from the source. The parameter
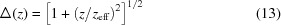
is the expansion coefficient and
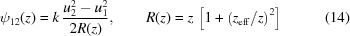
are the phase and radius of curvature of the GSM beam. In (13) and (14) the effective distance
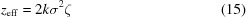
has been introduced (Gbur & Wolf, 2001 ▶; Vartanyants & Singer, 2010 ▶). At that distance the expansion coefficient is equal to 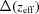 = 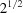. In the limit of a spatially coherent source, 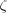 = 1, the effective distance 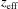 coincides with the Rayleigh length 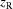 = 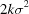, which is often introduced in the theory of optical Gaussian beams (Saleh & Teich, 1991 ▶). It is noteworthy that the CSD of the beam downstream of the source is not homogeneous, *i.e.* 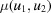 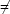 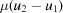 due to the phase factor 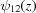 in (11).

It is important to note here that in the frame of the GSM the coherence properties of the beam at any position along the beamline containing OEs (see Fig. 1 ▶) will be described by the same equation (11) with different meaning of the parameters 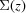, 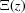, 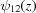 and 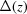.

The global degree of coherence of a GSM source can be expressed as (Vartanyants & Singer, 2010 ▶)
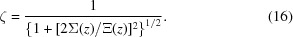
One important property of the GSM beams is that in the case of free space propagation the global degree of coherence remains constant [see equations (12) and (16)].

### 2.3. Propagation through optical elements

The propagation of the CSD through a thin OE can be described by a complex valued transmission function 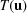 (Goodman, 1985 ▶),
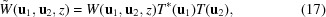
where 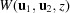 and 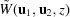 are the CSDs incident on and just behind the OE. It is interesting to note that a thin OE described by a transmission function 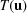 does not change the transverse coherence properties in its plane. It can be readily seen from (5) and (17) that the modulus of the SDC in front of 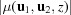 and behind 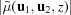 the lens are the same, 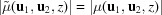. This also implies that according to (11) the coherence length 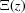 will be preserved.

In general, the propagation of partially coherent radiation through a beamline with a thin OE can be performed in the following steps. First, the CSD 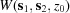 at the source is defined. The propagation of the CSD 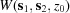 from the source to the first OE positioned at 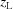 can be described by equations (7) and (11). For the propagation of the CSD, 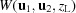 through the OE equation (17) can be utilized. Finally, the coherence properties at any position 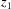 downstream of this OE are obtained using (7). The extension of this procedure to simulate the propagation of partially coherent radiation through a beamline containing several OEs is straightforward, provided each OE can be described well in the frame of the thin OE approximation. Below we will implement this scheme for a simple beamline geometry containing an undulator source described by a plane GSM source and a focusing element positioned at a distance 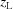 downstream of the source (see Fig. 1 ▶).

## 3. Focusing of partially coherent X-ray beams

### 3.1. Compound refractive lenses

As a focusing element we will consider a parabolic CRL (Lengeler *et al.*, 1999 ▶). The complex valued transmission function 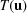 of such a lens can be written in the form^4^

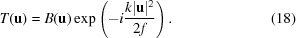
The function 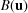 defines the absorption and opening aperture of the lens and *f* is its focal length (Saleh & Teich, 1991 ▶; Goodman, 2005 ▶),
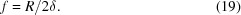
Here δ is the real part of the complex index of refraction (Born & Wolf, 1999 ▶) *n* = 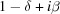 that is of the order of 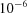 for X-ray energies. The parameter β is the imaginary part of the refractive index and describes absorption. Since δ is extremely small for X-rays, typically several lenses are stacked together (see Fig. 1 ▶) to reduce the focal length and improve the focusing properties of the lenses. For a combination of *N* lenses the focal length is given by 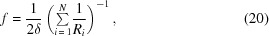
where 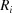 is the radius of *i*th lens. The above expression holds if the total arrangement of lenses fulfils the thin-lens approximation.

Lens imperfections or aberrations, if present, can be taken into account by introducing additional phase factors in 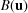. Here, we restrict ourselves to aberration-free optics and assume that for a thin parabolic lens the opening aperture function can be described by a Gaussian function,
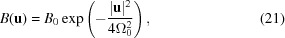
where 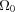 is the effective opening aperture due to absorption in the material of the lens defined through 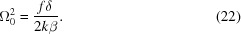
The parameter 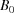 describes the transmission of the lens in its center and satisfies 0 < 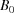 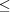 1.

It is important to note that often the opening aperture of the OE is determined not by the natural absorption but rather by the size of the lens or beam-defining aperture in front of the lens. When this additional aperture 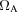 is comparable with or smaller than the effective aperture 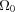 we introduce the total aperture 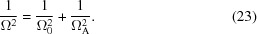
To simplify the analysis we consider here a Gaussian form of the additional aperture. We show in Appendix *A* [App appa] that the coherence properties of the focused radiation do not significantly change if a rectangular aperture of the corresponding size is used.

### 3.2. Propagation of Gaussian Schell-model beams through focusing elements

**Figure 2:**
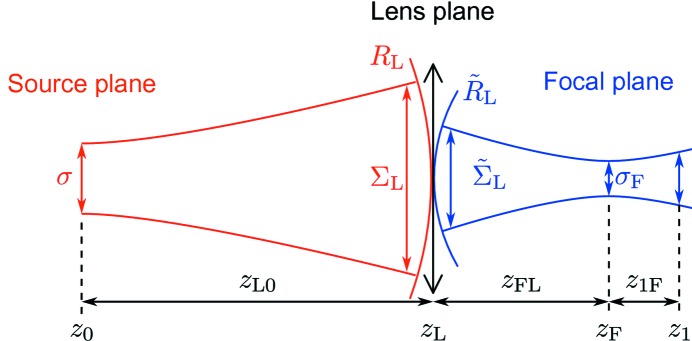
The propagation geometry. The partially coherent source with a source size σ and coherence length ξ is positioned at 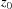. Partially coherent radiation with the beam size 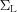, coherence length 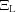 and radius of curvature 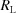 is incident on the lens at 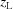. The beam size 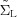 and the radius of curvature 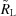 are modified by the transmission through the lens. The beam is focused at the focal position 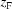 with the focus size 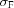 and coherence length ξ_F_. The beam parameters at an arbitrary position 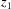 downstream of the lens are determined.

To simulate propagation of partially coherent radiation through a focusing element we will use the procedure outlined above (see Fig. 2 ▶). The source at 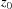 will be described in the frame of the Gaussian Schell-model with the CSD 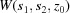 defined in equations (9) and (10). The CSD function incident on the lens at 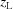 is given by (11). The parameters 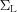 = 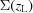, 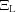 = 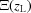, 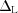 = 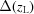 and 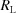 = 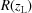 are the beam size, transverse coherence length, expansion coefficient and radius of curvature incident on the lens at 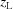, respectively [see equations (12), (13) and (14)]. Substituting the lens transmission function introduced in equations (18) and (21) into equation (17) we can determine the CSD immediately behind the lens. The beam behind a Gaussian lens with an opening aperture Ω can be again discribed by the GSM using (11) with the modified beam size
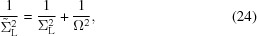
radius of curvature
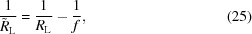
and normalization constant 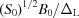 (see Fig. 2 ▶). As mentioned earlier, in the thin-lens approximation the coherence length 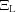 = 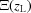 is not modified while transmission of the incident beam through the lens.

If the beam size 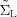 is reduced due to a finite aperture Ω of the lens the global degree of transverse coherence (16) behind the lens can be defined as
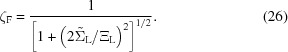
For partially coherent Gaussian beams this value will be constant at all positions downstream of the lens.

**Figure 3:**
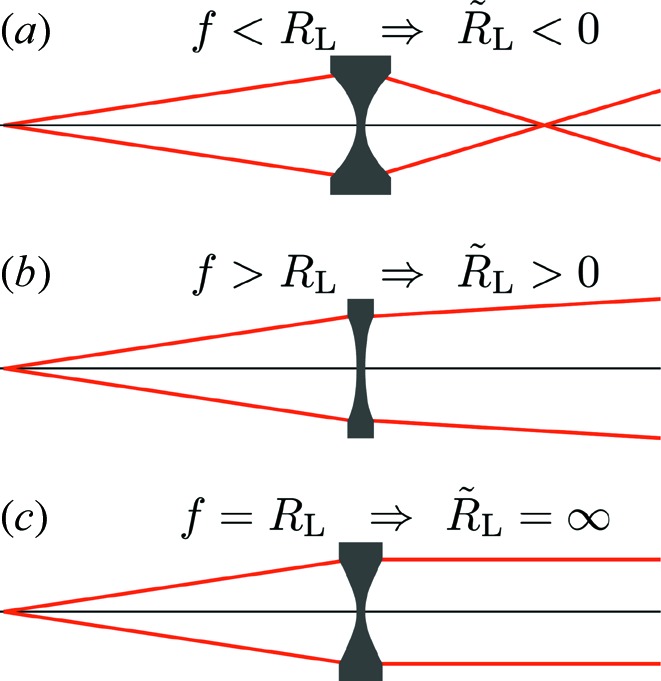
Different focusing geometries. (*a*) The beam is focused. (*b*) The divergence of the beam is reduced, but the beam is not focused. (*c*) The beam is collimated.

It is well known that the focusing properties of a lens are determined by the focal length *f*. Depending on the sign of *f*, the lens acts as a focusing (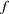 > 0) or a defocusing (*f* < 0) optical element. We consider a lens with the focal length 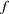 > 0, which according to (25) reduces the radius of curvature of the incident beam 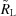. If the focal length is smaller than the curvature of the incident beam, *f* < 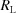, then according to (25) the radius of curvature behind the lens 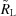 is negative and the beam is focused downstream of the lens (see Fig. 3*a* ▶). In the opposite case of *f* > 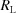, equation (25) yields a positive radius of curvature 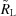 behind the lens. The divergence of the beam is reduced; however, the beam is not focused and a virtual focus lies upstream from the lens (see Fig. 3*b* ▶). If *f* = 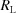 the radius of curvature behind the lens is infinite, which means that the beam is collimated (see Fig. 3*c* ▶).

### 3.3. Coherence properties of the beam behind the focusing element

To determine the beam properties in the focal plane one can apply the general propagation formula (7) to the radiation immediately behind the lens. However, it is more convenient to use the optics reciprocity theorem (Born & Wolf, 1999 ▶). We assume that a source is located at the focal position 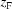 and the beam is characterized by its CSD in the frame of the GSM by equations (9) and (10), with its source size and coherence length in the focus given by the parameters 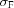 and 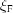, respectively. To calculate these parameters we propagate partially coherent beam from the focal position 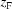 backwards to the lens position 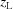 using equation (11) and compare it with the CSD function corresponding to the radiation transmitted through the lens. The parameters of the focus satisfying this boundary condition are given by (see Appendix *B* [App appb] for details)
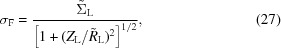


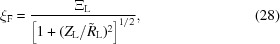
where we introduced 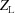 = 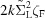, which is similar to the effective distance 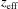 defined in equation (15).

The distance 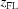 from the lens to the focus is given by (see Appendix *B* [App appb] for details)
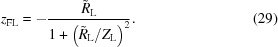
In this model the radius of curvature of the radiation in the focus is infinitely large and the phase 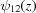 term in equation (11) vanishes. It is readily seen from equations (25) and (29) that the focal position coincides with the focal length of the lens, 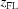 = *f*, only if the radius of curvature 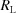 incident on the lens is much larger than the focal length, 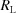 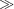 *f* and 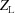 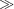 *f*, that is typically the case for the third-generation X-ray synchrotron sources.

The depth of focus 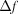 is the region along the optical axis where the beam size is smaller than the focus size multiplied by 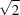. It is typically defined through the Rayleigh length for coherent Gaussian beams (Saleh & Teich, 1991 ▶) and can be extended to partially coherent beams introducing the effective distance 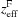 = 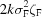 in the focus,
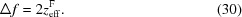
After the position of the focus and transverse coherence properties in the focus have been obtained, it is possible to calculate the CSD at any position 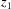 downstream of the lens applying equations (11)–(15). In these equations the source size σ, transverse coherence length at the source ξ and the global degree of coherence ζ are replaced by the values 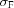, 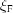 and 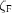 in the focus from equations (26), (27) and (28). The distance *z* from the source to the observation plane is replaced by 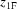 = 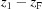, which is the distance between the observation plane at 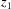 and the focus at 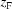 (see Fig. 2 ▶). Below, the limits of a fully coherent or diffraction-limited focus as well as a rather incoherent focus will be discussed.

## 4. Diffraction-limited focus

We will consider now a strongly focusing lens, which substantially increases the flux density in the focus and is especially interesting for practical applications. According to equation (27) a small focal size 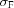 occurs when the denominator in equation (27) is large. This is equivalent to the condition that the beam curvature behind the lens 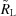 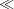 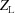. In this limit we obtain from equation (27)

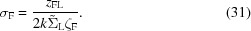
Here we also used the fact that in the same limit of 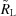 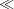 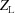 according to equation (29) the focal distance 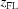 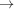 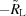 and can be expressed as 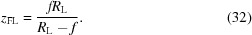
Introducing the diffraction-limited focus size 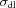 = 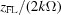, equation (31) can be presented as 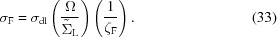
The diffraction limit can be equivalently written as 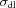 = 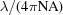, where NA = 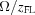 is the numerical aperture of the lens.

**Figure 4:**
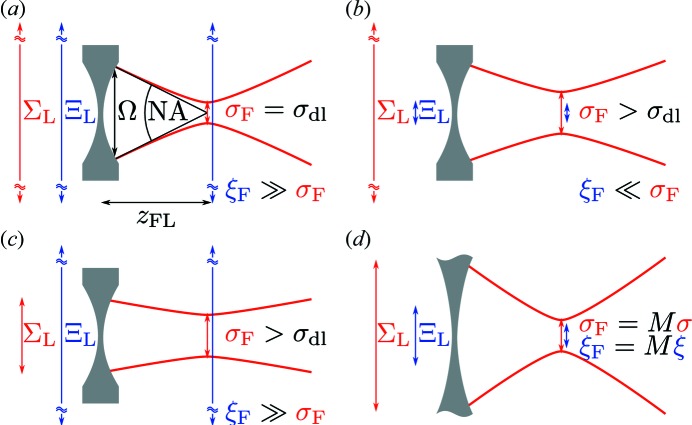
(*a*) Focus is diffraction-limited 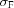 = 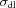 if the beam size 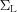 and transverse coherence length 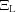 are larger than the lens aperture Ω. (*b*, *c*) Focus size is larger than the diffraction limit if the coherence length (*b*) or beam size (*c*) incident on the lens is smaller than the aperture. (*d*) For a very large aperture the focus is a demagnified image of the source.

Using the concept of the diffraction limit [see equation (33)], important cases for focusing of partially coherent radiation can be identified. It can be immediately seen from equations (24) and (33) that the diffraction limit is the smallest possible focus size achievable with the lens, since 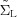 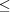 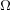 and 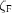 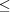 1 by definition. It is also clear from equations (24), (26) and (33) that the focus is diffraction-limited only if both the beam size and transverse coherence length of the beam incident on the lens are much larger than the lens aperture [see Fig. 4(*a*) ▶]. The focus size increases if either the beam size or coherence length of the beam incident on the lens is smaller than the aperture of the lens [see Figs. 4(*b*) and 4(*c*) ▶]. However, there is an important difference between these two cases. In the first example a highly coherent beam is obtained in the focus and the blurring of the focus size is due to diffraction effects of a finite incoming beam [see Fig. 4(*b*) ▶]. In the second case the beam in the focus is rather incoherent and the larger focus is a consequence of a small degree of coherence in the focus [see Fig. 4(*c*) ▶].

**Figure 5:**
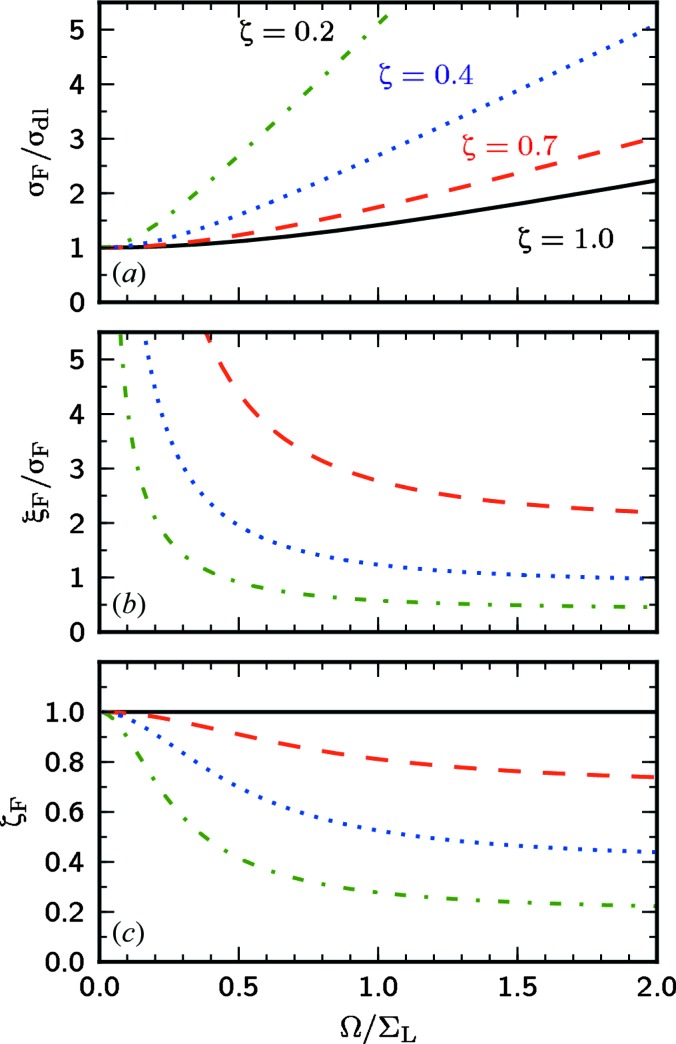
The normalized focus size 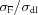 (*a*), transverse coherence length 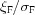 (*b*), and global degree of coherence 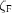 in the focus (*c*) as functions of the ratio 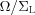. Different values of the global degree of coherence at the source 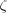 = 1.0, 0.7, 0.4 and 0.2 are considered.

We can also express the focus size in terms of the beam parameters incident on the lens, which may be important for practical purposes. Substituting (24) and (26) into (33), we can obtain 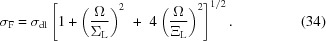
The focus size as a function of the source size and the lens aperture is presented in Appendix *C* [App appc]. The coherence length in the focus can be calculated using the ratio 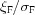 = 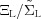 [see equations (27), (28) and (24)] and 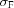 from (34),
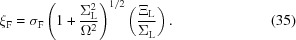
We demonstrate the obtained results in Fig. 5 ▶, where the focal size 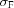 (27), coherence length 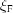 (28) and degree of coherence 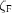 (26) in the focus are calculated as a function of the ratio 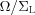 for different values of the degree of coherence ζ of the incoming beam. It can be clearly seen from this figure that a diffraction-limited focus size is obtained in the limit of a fully coherent beam. With the reduced coherence of the incident beam the focal size is increased rapidly. At the same time for the smaller apertures the diffraction limit can be reached for beams of any degree of coherence; however, at the expense of limited photon flux. Even for a highly coherent beam, the focus is larger than the diffraction limit if the beam size is smaller than the lens aperture. As can be clearly seen from Fig. 5(*b*) ▶ the ratio of the transverse coherence length to the focus size 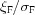 increases rapidly and approaches infinity for smaller apertures. As the focal size approaches the diffraction limit the degree of coherence approaches the fully coherent value of 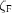 = 1 at small apertures [see Fig. 5(*c*) ▶].

A summary of equations obtained in this section to determine the beam properties in the focus is given in Table 1 ▶.

## 5. Focusing element with a large aperture

If the lens aperture Ω is significantly larger than the beam size of the incident radiation 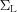, the lens only modifies the radius of curvature. Then the beam size and coherence length in the focus can be expressed through the same parameters at the source through simple relations (Turunen & Friberg, 1986 ▶)^5^

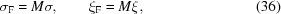
where
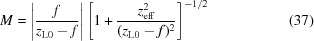
is the magnification factor [see Fig. 4(*d*) ▶]. The ratio between the transverse coherence length and the beam size is constant everywhere along the optical axis and is determined by the source parameters 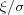. The same holds for the degree of transverse coherence ζ. As an important result we note that in the frame of the GSM the focus generated by a CRL with a sufficiently large aperture is just a scaled image of the source.

In the limit of geometrical optics, when diffraction effects can be neglected and the degree of coherence approaches zero 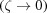 (Born & Wolf, 1999 ▶; Goodman, 2005 ▶), the effective distance vanishes, 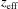 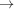 0, and the magnification factor simplifies to
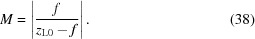
The same limit is approached if the distance 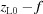 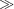 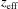, which is typical for synchrotron sources. A summary of the equations applicable for the case of large apertures is presented in Table 2 ▶.

## 6. Focusing of X-ray beams at third-generation synchrotron sources

We have applied the general approach developed in the previous sections to simulate the coherence properties of the focused X-ray beams at the beamline P10 at PETRA III. This beamline is dedicated to coherence applications such as CXDI and XPCS and understanding of the coherence properties in the focus is vital for the success of these experiments.

As an example we analyzed an optical system installed at this beamline, which consists of three berylium CRLs with radii of 200 µm, 50 µm and 50 µm and is positioned at a distance of 85 m downstream of the source (Zozulya *et al.*, 2012 ▶). We considered this set of lenses as a thin lens and applied equations (20) and (23) to determine the focal length 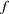 = 2.13 m and the effective aperture of the lens due to absorption 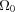 = 242 µm. The geometrical size of the 50 µm lenses is 450 µm.^6^

We analyzed the coherence properties of such a lens as a function of the aperture size 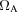. To determine the beam properties in the region near the focal plane we have used the general expression (11). The parameters of the source were considered for a photon energy of 8 keV and low-β operation of the synchrotron source (see Table 3 ▶). It is immediately seen that the radiation in the horizontal and vertical directions can be considered as incoherent and coherent, respectively (see also Fig. 5 ▶). Equation (23) was used to calculate the total aperture size Ω of the focusing element including the beam-defining aperture. Aperture sizes 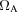
 (Ω) of 25 (25) µm and 100 (93) µm were considered in the horizontal and 50 (49) µm and 150 (128) µm in the vertical direction (see Table 4 ▶).

**Figure 6:**
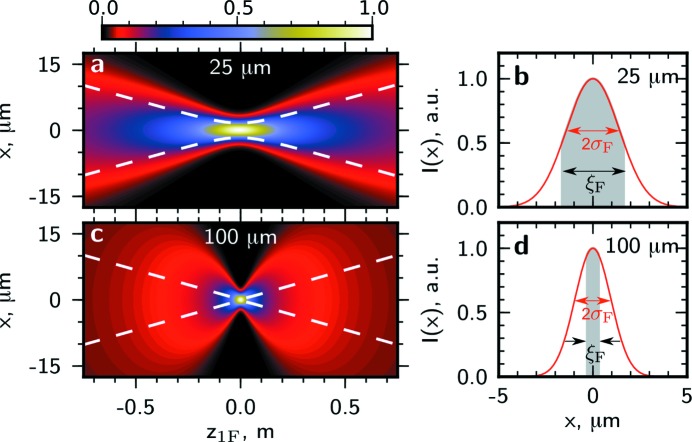
The intensity profile in the vicinity of the focus as a function of the propagation distance 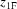 for the aperture sizes 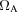 of 25 µm (*a*), 100 µm (*c*) in the horizontal direction. The white dashed lines indicate the coherent part of the beam with a width given by the transverse coherence length. (*b*), (*d*) Line scans of the intensity profile 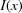 from (*a*), (*c*) in the focal plane at 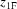 = 0. The shaded region shows the coherent part of the beam with the width corresponding to the transverse coherence length 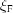 in the focal plane.

**Figure 7:**
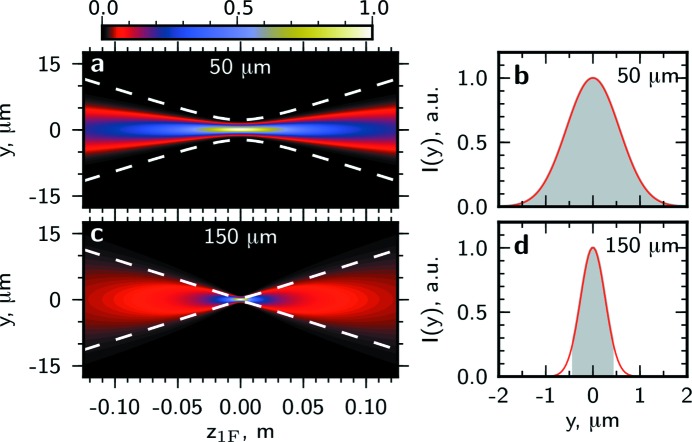
The same as in Fig. 6 ▶ in the vertical direction and for the aperture sizes 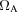 of 50 µm (*a*), (*b*), 150 µm (*c*), (*d*).

In Figs. 6 ▶ and 7 ▶ the intensity profile and transverse coherence properties at different distances from the lens around the focal position in the horizontal and vertical directions are presented. In the horizontal direction for an aperture size of 25 µm the coherence length is about two times larger than the beam size and the beam is highly coherent [see Figs. 6(*a*) and 6(*b*) ▶]. The depth of focus is about 10 cm. For a significantly larger aperture size of 100 µm the focus size and the depth of focus are smaller and the beam coherence is poor [see Figs. 6(*c*) and 6(*d*) ▶]. In the vertical direction the beam size and the depth of focus are significantly smaller than for the horizontal direction (note the different scales in Figs. 6 ▶ and 7 ▶). Due to a higher degree of coherence at the source in the vertical direction, highly coherent radiation in the focus can be achieved with larger apertures. For the aperture size of 50 µm the coherence length is significantly larger than the beam size and the beam is fully coherent [see Figs. 7(*a*) and 7(*b*) ▶]. Even for a comparably large aperture of 150 µm the coherence length substantially exceeds the beam size [see Figs. 7(*c*) and 7(*d*) ▶].

**Figure 8:**
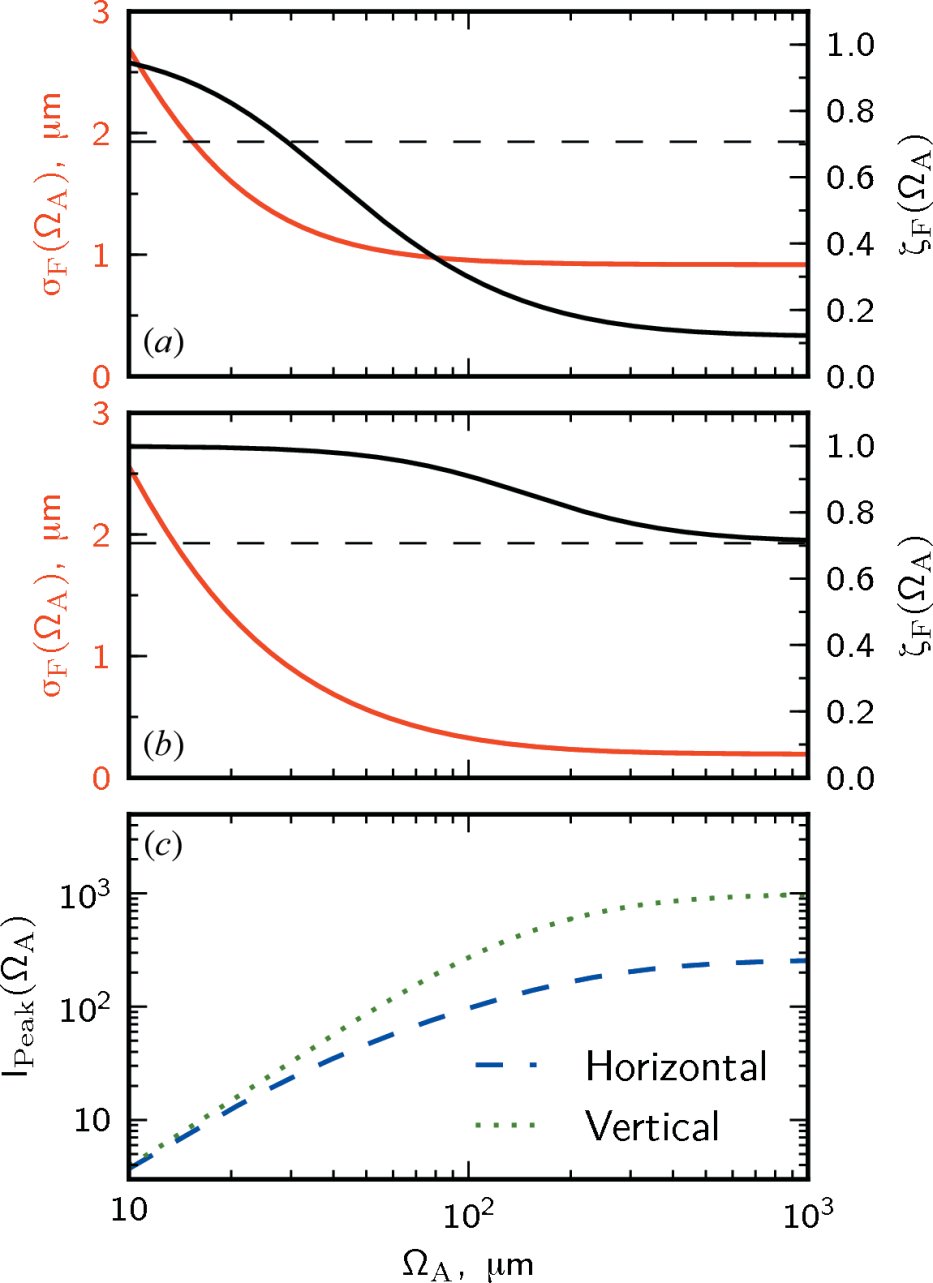
The global degree of coherence (black line) and the focal size (red line) as functions of the beam-defining aperture size 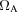 in the horizontal (*a*) and vertical (*b*) directions. The dashed line indicates a highly coherent beam with the coherence length in the focus being twice as large as the r.m.s. beam size, 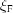 = 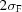. (*c*) The increase in the flux density 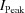 as a function of the beam-defining aperture size in the horizontal (blue dashed line) and vertical (green dotted line) directions.

For coherence-based applications the most important properties of an X-ray lens are the small focus size, increase of the degree of coherence in the focus and increase in the peak intensity 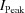 = 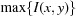. These quantities as functions of the opening aperture size 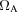 are presented in Fig. 8 ▶ for the horizontal and vertical directions.^7^ As can be seen in Figs. 8(*a*) and 8(*b*) ▶, at the largest apertures both the focus size and degree of coherence have constant values and the smallest focus size is obtained. The focus size 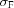 is increased at smaller aperture values 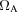 due to diffraction as described in §4. At the same time the degree of coherence 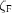 reaches its maximum value close to 1. In the horizontal direction it increases from a value of 10% with large beam-defining aperture 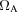 to 71% [horizontal dashed line in Fig. 8(*a*) ▶] for an aperture size of about 30 µm. This can be considered as a highly coherent beam with the coherence length being twice the size of the beam. In the vertical direction the degree of coherence is higher than 71% for all aperture sizes of the optical system considered here. The peak intensity in the focus theoretically can be increased by more than two orders of magnitude in both directions for large apertures [see Fig. 8(*c*) ▶]. At the same time, for the small aperture sizes the amount of the total transmitted flux is reduced. In this focusing geometry using an aperture size of 30 µm (H) × 100 µm (V) a highly coherent beam with a focus size of 1.2 µm (H) × 0.3 µm (V) is expected. In this case 0.2% of the total flux is transmitted through the lens and the flux density is increased by three orders of magnitude.

We have compared the results of our approach with the measurements of the beam size performed at the coherence beamline P10 (Zozulya *et al.*, 2012 ▶). A transfocator with seven 50 µm berylium CRLs was used at an energy of 13.2 keV. The beam-defining slits were set to 100 µm in both directions and a focus size (FWHM) of 2.9 µm (H) × 2.9 µm (V) was observed. Applying our approach for the same lens parameters (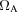 = 22) and the estimated source properties at a photon energy of 13.2 keV (Vartanyants & Singer, 2010 ▶) yields a theoretical focus size (FWHM) of 2.4 µm (H) × 1.5 µm (V). Our simulations reproduce well the experimental focus size in the horizontal direction; however, they are about twice as small as the measured values in the vertical direction. This can be attributed to the fact that all optical components at P10 deflect the beam in the vertical direction and we expect the deviation of the experimental and theoretical values to be larger in this direction.

## 7. Conclusions

We have presented an analytic approach to propagate partially coherent X-ray beams through focusing elements, which is based on the results of statistical optics and can be applied to X-ray beams at third-generation synchrotron sources. As an example, parabolic compound refractive lenses were analyzed in detail. The same formalism can also be applied to Fresnel zone plates and other focusing optics, which can be treated within the thin-lens approximation. We have obtained simple equations for the case of a strongly focusing lens. Since the method is analytical it can be effectively used to estimate the beam parameters at the experimental station. Important limiting cases, such as rather coherent and in­coherent radiation, have been considered, which represent synchrotron radiation in the vertical and horizontal directions, respectively. As an example we have performed calculations for the coherence beamline P10 at the PETRA III storage ring. We anticipate that our approach can also be applied to estimate the performance of focused beams at highly coherent X-ray free-electron laser sources. 

## Figures and Tables

**Figure 9:**
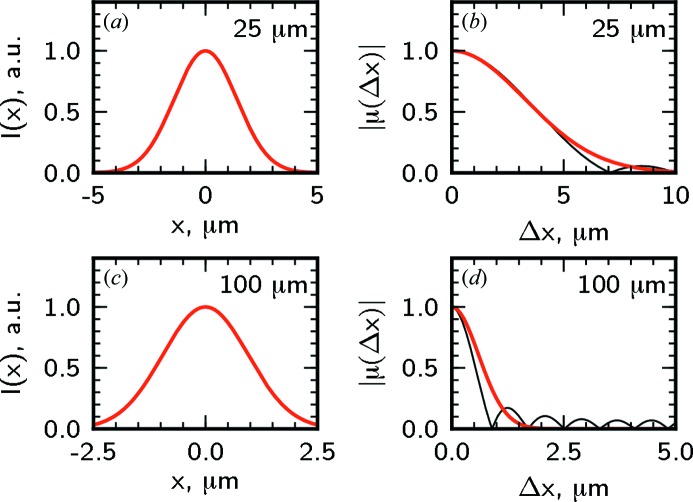
The intensity profile 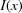 (*a*), (*c*) and modulus of the spectral degree of coherence 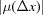 (*b*), (*d*) in the focal plane in the horizontal direction. Calculations made for aperture sizes of 25 µm (*a*), (*b*) and 100 µm (*c*), (*d*) with a Gaussian aperture (red lines) and a slit (black line) are presented.

**Figure 10:**
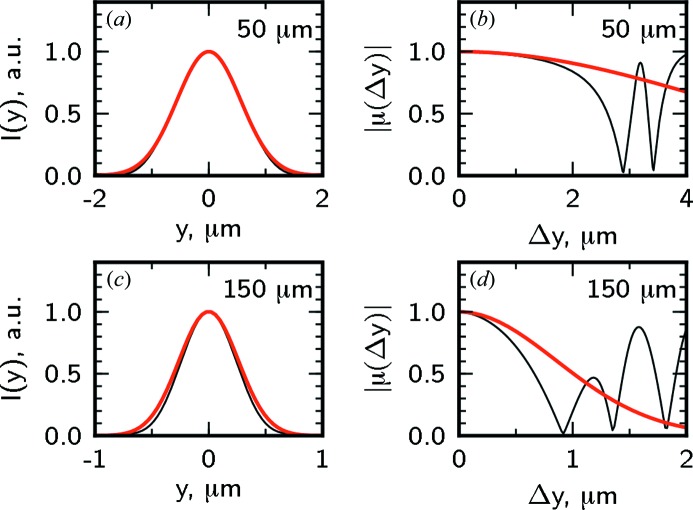
The same as in Fig. 9 ▶ in the vertical direction for aperture sizes of 50 µm (*a*), (*b*) and 150 µm (*c*), (*d*).

**Table 1:** Coherence properties in the focus of a strongly focusing lens 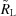 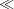 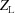 with an arbitrary lens aperture

Focus size	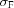 = 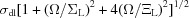
Diffraction limit	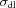 = 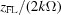
Transverse coherence length	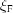 = 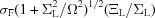
Focus position	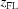 = 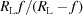
Depth of focus	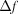 = 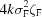

**Table 2:** Coherence properties in the focus of a lens with an aperture much larger than the beam size, 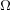 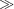 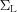

Focus size	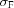 = 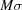
Transverse coherence length	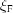 = 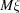
Focus position	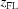 = 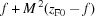
Depth of focus	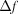 = 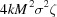
Magnification	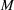 = 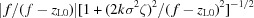

**Table 3:** Beam parameters of the PETRA III source (low-β) for a photon energy of 8 keV (Balewski *et al.*, 2004 ▶) The coherence properties at the source and at a distance of 85 m downstream of the source are presented (Vartanyants & Singer, 2010 ▶).

	Horizontal	Vertical
Beam size at the source (µm)	36.2	6.3
Transverse coherence length at the source (µm)	0.9	7.7
Beam size at 85 m (µm)	2370	320
Transverse coherence at 85 m (µm)	58	390
Degree of coherence ζ	0.01	0.52

**Table 4:** Coherence properties in the focus of the beamline P10 at PETRA III calculated for different apertures 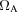 in front of the lens

	Horizontal		Vertical	
Aperture size 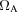 (µm)	25	100	50	150
Total aperture size Ω (µm)	25	93	49	128
Focus size 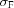 (µm)	1.4	1.0	0.6	0.3
Transverse coherence length 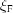 (µm)	3.3	0.6	4.5	0.9
Global degree of coherence 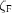	0.76	0.30	0.97	0.85
Depth of focus 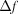 (mm)	120	22	25	3.6

## Footnotes

1 Here we restrict ourselves to stationary radiation fields, which are generated at third-generation synchrotron sources.

2 In this equation and below we omit the frequency dependence ω for brevity. The GSM will be applied to narrow-bandwidth radiation, where ω is the average frequency.

3 It is noteworthy that the CSD of a GSM source can be factorized into two transverse components. We will present calculations for one transverse direction and will drop the subscript for brevity.

4 In principle, the transmission function can depend on the frequency of radiation ω. However, for CRLs in the X-ray range for a bandwidth lower than  the frequency dependence can be neglected (Kohn, 2012 ▶).

5 These expressions hold even without the strong lens approximation used earlier in the paper and can be obtained from equations (27) and (28) by a straightforward calculation (Singer, 2012 ▶).

6 We should note that the geometrical lens size limits the aperture . According to our estimates it corresponds to an r.m.s. width of about 100 µm (see Appendix *A* [App appa]).

7 For this set of lenses, aperture sizes of more than 100 µm are larger than the geometrical size of the lens and are shown to illustrate the asymptotic behaviour.

## Appendix A Edge effects of a beam-defining slit on the coherence properties in the focus

A potential problem of the application of the Gaussian beam-defining aperture to the case of synchrotron radiation sources is the fact that at most beamlines a slit or a pinhole is used, which has a non-Gaussian transmission function. To understand how significant the edges of such apertures can be, we performed numerical propagation of the CSD from the lens with an aperture in the form of a slit with the size *D* [the case of the pinhole was considered by Singer & Vartanyants (2011 ▶)]. Equation (7), (8) and (17) were solved numerically using the following transmission function of the optical system,

where  = 1 if  < *D*/2 and 0 elsewhere. The lens aperture due to absorption  and the focal length of the lens *f* were the same as in the main text. The slit transmission function  was convolved by a Gaussian with a width of 20 µm, to smooth the hard edges. These simulations were compared with our analytical approach. The size of the Gaussian aperture  was related to the slit size *D* by a comparison of the intensity profiles (FWHM) generated by a Gaussian and a rectangular transmission function. The best match was found for the condition *D* = .

In Figs. 9 ▶ and 10 ▶ the intensity profile and the spectral degree of coherence in the focus are shown for the same aperture sizes as in Figs. 6 ▶ and 7 ▶. For comparison the corresponding intensity profiles and SDC obtained in numerical simulations are shown in the same figures. It can be clearly seen that the beam profile determined through the analytical model presented in this work coincides well with the results of numerical simulations. Apart from oscillations in low-intensity regions, which appear due to diffraction on hard edges of the slit, the Gaussian model describes well the coherence properties of the focused beam in the horizontal direction. In the vertical direction the edge effects are substantial due to a high coherence, and the GSM slightly overestimates the coherence length of the beam.

## Appendix B Derivation of the focal distance, focus size and transverse coherence length

To determine the radiation properties in the focus we use the optics reciprocity theorem. We consider the focus as a GSM source and the radiation behind the lens as back propagated from the focus. We have to determine the focus size , transverse coherence length  and distance from the lens to the focus  from the beam size , coherence length  and radius of curvature  immediately behind the lens. These parameters are connected through the expressions for the expansion coefficient , (11) and (12),

and the radius of curvature, (14),

To solve this set of equations we rewrite the expansion coefficient  in terms of the radius of curvature,

Introducing a new variable  =  = , which can be calculated from the parameters of the incident radiation and the lens aperture using equations (24), (26) and (42), we rewrite

and

Now substituting (43) into (44) we find the distance from the lens to the focus,

Substituting (45) into (43) we find the expansion coefficient,

## Appendix C Focus size as a function of source size

Here we show that in the strong focusing approximation the focus size can be given as a convolution of the diffraction limit  and demagnified source size , where *M* is the demagnification factor. Substituting the definition of the diffraction limit  =  into (34) we find

Now, using equations (12), (13), (15) and (16) in the far-field approximation   , we find

The focus size can then be given by

with the demagnification factor expressed as *M* = . In the strong focusing approximation    using equations (25) and (29) the demagnification factor can be given as

It is interesting to note that, when the beam is incoherent,   , and the aperture is larger than the transverse coherence length,   , we find from equation (34)  =  [see Figs. 5 ▶ and 4(*b*) ▶]. Under these conditions the focus size is determined only by the transverse coherence length of the beam incident on the lens. Rewriting the condition for the focus size as  =  it can readily be seen that this case is very similar to the van Cittert–Zernike theorem (Mandel & Wolf, 1995 ▶). The focus can be considered as a planar incoherent GSM source and the transverse coherence length at a distance  from this source is given by . In fact, the coherence length is demagnified in the focus by the lens.
